# Diagnostic Worth of Mallein and Tuberculin

**Published:** 1897-05

**Authors:** 


### ABSTRACTS FROM FOREIGN JOURNALS.

### GERMAN.

UNDER THE DIRECTION OF J. PRESTON HOSKINS,
PRINCETON, N. J.

DIAGNOSTIC WORTH OF MALLEIN AND TUBERCULIN. Prof. Semmer gives the result of the numerous tests of mallein which were made under his direction during the year 1893–'94. In all 952 horses were subjected to the test, and in 561 of these a rise in temperature of 2° to 3.5° C. manifested itself. All of the 391 cases which did not react remained well, and in the few cases where post-mortems were held no symptoms of glanders (strangles) could be detected. In the 157 cases in which a rise of temperature took place post-mortems were also held on two animals without result; in all the others, not a single one of which showed external symptoms of the disease, the specific lesions were found; they were not, however, very characteristic: swelling of some of the lymph-glands internally with indurations, little ulcers, thickening of the mucous membrane of the nose, etc. In almost all cases fresh, soft, gray-colored granulations were found in the lungs, which sometimes were in a condition of cheesy degeneration. It often costs much trouble and care to trace out the pathological changes. These characteristic symptoms and the frequency of their occurrence, especially in southern Russia, led Semmer to the conclusion that this form of the disease was a mild strangles. The animals frequently recovered, and the bacteriological investigations gave, for the most part, only negative results. Semmer regards mallein as the surest means of diagnosis in strangles, rendering it possible to detect the disease in its mildest and most latent form. He considers tuberculin also as a valuable diagnostic means, and recommends its general use.—*Veeartsenij. Bladen*, Deel ix. Heft 4.

